# STAT1 Isoforms Differentially Regulate NK Cell Maturation and Anti-tumor Activity

**DOI:** 10.3389/fimmu.2020.02189

**Published:** 2020-09-11

**Authors:** Katrin Meissl, Natalija Simonović, Lena Amenitsch, Agnieszka Witalisz-Siepracka, Klara Klein, Caroline Lassnig, Ana Puga, Claus Vogl, Andrea Poelzl, Markus Bosmann, Alexander Dohnal, Veronika Sexl, Mathias Müller, Birgit Strobl

**Affiliations:** ^1^Institute of Animal Breeding and Genetics, University of Veterinary Medicine Vienna, Vienna, Austria; ^2^Institute of Pharmacology and Toxicology, University of Veterinary Medicine Vienna, Vienna, Austria; ^3^Biomodels Austria, University of Veterinary Medicine Vienna, Vienna, Austria; ^4^Pulmonary Center, Department of Medicine, Boston University School of Medicine, Boston, MA, United States; ^5^Center for Thrombosis and Hemostasis, University Medical Center Mainz, Johannes Gutenberg University Mainz, Mainz, Germany; ^6^Tumor Immunology, St. Anna Kinderkrebsforschung, Children’s Cancer Research Institute, Vienna, Austria

**Keywords:** NK cells, interferon, signal transduction, isoforms, IL-15Rα, MHC class I

## Abstract

Natural killer (NK) cells are important components of the innate immune defense against infections and cancers. Signal transducer and activator of transcription 1 (STAT1) is a transcription factor that is essential for NK cell maturation and NK cell-dependent tumor surveillance. Two alternatively spliced isoforms of STAT1 exist: a full-length STAT1α and a C-terminally truncated STAT1β isoform. Aberrant splicing is frequently observed in cancer cells and several anti-cancer drugs interfere with the cellular splicing machinery. To investigate whether NK cell-mediated tumor surveillance is affected by a switch in STAT1 splicing, we made use of knock-in mice expressing either only the STAT1α (*Stat1*^α/α^) or the STAT1β (*Stat1^β^^/^^β^*) isoform. NK cells from *Stat1*^α/α^ mice matured normally and controlled transplanted tumor cells as efficiently as NK cells from wild-type mice. In contrast, NK cells from *Stat1*^β/β^ mice showed impaired maturation and effector functions, albeit less severe than NK cells from mice that completely lack STAT1 (*Stat1^–/–^*). Mechanistically, we show that NK cell maturation requires the presence of STAT1α in the niche rather than in NK cells themselves and that NK cell maturation depends on IFNγ signaling under homeostatic conditions. The impaired NK cell maturation in *Stat1*^β/β^ mice was paralleled by decreased IL-15 receptor alpha (IL-15Rα) surface levels on dendritic cells, macrophages and monocytes. Treatment of *Stat1*^β/β^ mice with exogenous IL-15/IL-15Rα complexes rescued NK cell maturation but not their effector functions. Collectively, our findings provide evidence that STAT1 isoforms are not functionally redundant in regulating NK cell activity and that the absence of STAT1α severely impairs, but does not abolish, NK cell-dependent tumor surveillance.

## Introduction

The Janus kinase (JAK)/signal transducer and activator of transcription (STAT) pathway is employed by many different cytokines and regulates diverse cellular processes, such as differentiation, proliferation, and survival (1). JAKs are receptor-associated tyrosine kinases that, upon ligand binding to receptor complexes, initiate a phosphorylation cascade that results in nuclear translocation of tyrosine phosphorylated homo- or heterodimeric STAT proteins to re-program gene expression (2). The STAT family of transcription factors consists of seven members, STAT1–4, STAT5A, STAT5B, and STAT6, that positively or negatively regulate natural killer (NK) cell activity (3, 4). STAT1 loss-of-function and gain-of-function mutations in humans lead to impaired NK cell cytotoxicity (4–7). Genetic ablation of *Stat1* in mice results in impaired NK cell maturation, cytotoxicity and NK cell-mediated tumor rejection, although underlying mechanisms are incompletely understood (8–12).

STAT1, STAT3, and STAT4 exist as two alternatively spliced isoforms: a full-length α-isoform and a C-terminally truncated β-isoform, which lacks the C-terminal transactivation domain (TAD). β-isoforms were initially believed to be transcriptionally inactive. However, studies in gene-modified mice challenged this concept by demonstrating that STAT1β, STAT3β, and STAT4β are capable of inducing gene expression that quantitatively and qualitatively differs from transcriptional responses induced by the respective α-isoform (13–16). Alternative splicing of STATs is evolutionarily conserved in mammals and also reported in fish (17, 18), suggesting that it is physiologically relevant. Pre-mRNA splicing is tightly regulated and represents a crucial layer of gene regulation in many processes, such as development, differentiation, and immunity. Splicing patterns are profoundly altered in cancer cells and evidence has accumulated that targeting the splicing machinery might be a promising strategy for cancer therapy (19–21). It is unknown how splicing of STAT1, STAT3, and STAT4 is regulated. Increasing evidence suggests that STAT3β acts as a tumor suppressor (22–24) and predominant expression of *STAT4*β mRNA has been reported in patients with inflammatory bowel disease (25). Murine and human cells contain predominantly STAT1α, although there has not been a thorough analysis of STAT1 isoform ratios in diverse cell types and under physiological and pathological conditions. Overexpression studies in cell lines indicated that STAT1 isoforms differ in their capacity to induce cell death in response to DNA-damaging agents (26, 27). More recently, high STAT1β levels have been associated with a good prognosis in oesophageal squamous cell carcinomas (28). Little is known about the role of the individual STAT1 isoforms in immunity *in vivo*. STAT1 isoforms are functionally redundant in the innate immune defense against type I or type III IFN-controlled viruses, such as encephalomyocarditis virus and rotaviruses, respectively. In contrast, STAT1β is less efficient than STAT1α in mediating the IFNγ-dependent innate immune defense against the intracellular bacterium *Listeria monocytogenes* and murine cytomegalovirus (16). The impact of STAT1 isoforms on tumor surveillance is unknown.

To assess the capacity of the individual STAT1 isoforms to promote NK cell maturation and anti-tumor activity, we used mice expressing either only STAT1α (*Stat1*^α/α^) or only STAT1β (*Stat1*^β/β^). We show that STAT1β is capable of driving NK cell maturation and effector functions, albeit less efficiently than STAT1α or both STAT1 isoforms. Mechanistically, we provide evidence that tonic STAT1α-dependent IFNγ signaling upregulates IL-15 receptor alpha (IL-15Rα) surface levels in accessory cells thereby facilitating late NK cell maturation under homeostatic conditions. Our data furthermore show that STAT1 has cell-intrinsic functions that promote NK cell cytotoxicity, which again are more efficiently exerted by STAT1α than STAT1β.

## Materials and Methods

### Mice

*Stat1^–/–^* [(*B6.129P2-Stat1^*tm1Dlv*^*, (29)], *Stat1*^α/α^ [(*B6.129P2-Stat1beta^*tm1Biat*^*, (16)], *Stat1*^β/β^ [(*B6.129P2-Stat1alpha^*tm1Biat*^*, (16)], *Ifnar1^–/–^* [(*B6.129P2-Ifnar1^*tm1Agt*^*, (30)], *Ifngr1^–/–^* [(*B6.129S7-Ifngr1^*tm1Agt*^*, (31)], and *Jak1^*fl/fl*^Ncr1Cre*[(*C57BL/6N-Jak1^*tm1c(EUCOMM)Hmgu/H*^*, (32)] mice were described previously. *Ifnar1^–/–^Stat1*^β/β^ and *Ifngr1^–/–^Stat1*^β/β^ mice were generated by crossing *Stat1*^β/β^ with *Ifnar1^–/–^* or *Ifngr1^–/–^* mice. *C57BL/6N* (wild-type, *WT*) mice were purchased from Janvier Labs, *Ly5.1* (*B6.SJL-Ptprc^*a*^*) from Charles River Laboratories and *Il27ra^–/–^* (*B6N.129P2-Il27ra^*tm1Mak/J*^*) from The Jackson Laboratory. β*2m^–/–^* [(β*2-microglobulin^–/–^*, *B6.129-B2m^*tm1Jae*^*, (33)] mice were kindly provided by Wilfried Ellmeier (Medical University of Vienna). All mice were housed under specific pathogen-free conditions according to FELASA guidelines at the animal facility of the Institute of Animal Breeding and Genetics, University of Veterinary Medicine Vienna, and were on *C57BL/6* background. Age-matched (6–9 weeks) animals were used for the experiments.

### Whole-Cell Protein Extracts and Western Blot

FACS-sorted splenic NK cells were lysed in a buffer containing 50 mM Tris/HCl pH 8.0, 10% (v/v) glycerol, 0.1 mM EDTA, 150 mM NaCl (all from Roth), 2 mM DTT, 0.5% NP40 (Igepal CA-630), 25 mM sodium fluoride, 0.2 mM sodium vanadate, 1 mM PMSF, 10 ng/μl leupeptin, 10 ng/μl aprotinin, 10 ng/μl pepstatin (all from Sigma-Aldrich) and lysates were cleared by centrifugation. Ten micrograms protein per lane were loaded and separated by SDS-PAGE and transferred onto nitrocellulose membranes (Hybond, GE Healthcare). As a molecular weight standard PageRuler^®^ Prestained Protein Ladder (Thermo Fisher Scientific) was used. Membranes were probed with anti-STAT1 (Cell Signaling Technology), anti-panERK (BD Transduction Laboratories) and IRDye fluorophor-conjugated secondary antibodies (LI-COR Biosciences). Blots were scanned with Odyssey^®^ Classic infrared imager (LI-COR Biosciences).

### Flow Cytometric Analysis and Antibodies

Spleen, blood, bone marrow, and liver single-cell suspensions (after erythrocyte lysis) were used to analyze surface markers by flow cytometry. Liver lymphocytes were isolated from perfused livers and were separated from hepatocytes by using 37.5% percoll (GE Healthcare). Spleen single-cell suspension was used to analyze transcription factors and cytokines by flow cytometry. For the analysis of surface IL-15Rα and STAT1, spleens were digested in medium containing collagenase D (1 mg/ml; Roche Applied Science) and DNase I (20 μg/ml; Roche Applied Science) prior to single cell isolation. The Foxp3/Transcription Factor staining Buffer Set (Thermo Fisher Scientific) or the fixation buffer and permeabilization wash buffer from BioLegend were used according to manufacturer’s instructions for fixation and permeabilization before intracellular staining of IFNγ, T-bet and of STAT1, respectively. The following antibodies were used: CD49b (DX5), NK1.1 (PK136), NKp46 (29A1.4), NKG2D (CX5), CD3ε (eBio500A2), KLRG1 (2F1), CD27 (LG.7F9), CD11b (M1/70), MHC class II (M5/114.15.2), CD11c (N418), MHC class I H-2Db (28-14-8), Ly49A (A1), Ly49 C/I (5E6), Ly49D (4E5), Ly49G2 (4D11), NKG2A/C/E (20d5), CD8 (53-6.7), CD3ε (17A2), B220 (RA3-6B2), F4/80 (BM8), Ly6G (1A8), Ly6C (HK1.4), IFNγ (XMG1.2), Ly5.2 (104), Ly5.1 (A20), T-bet (eBio4B10) from eBioscience/Thermo Fisher Scientific. Surface IL-15Rα was detected with a biotinylated antibody (R&D Systems) in combination with streptavidin APC (Thermo Fisher Scientific). Biotinylated IgG and streptavidin APC were used as isotype controls (R&D Systems and Thermo Fisher Scientific). The antibody for STAT1 detection and the isotype control were purchased from Miltenyi. Samples were acquired on a FACSCanto II (BD Bioscience) or on a CytoFLEX (Beckman Coulter) and analyzed using BD FACSDiva software version 8.0 or CytExpert version 2.2.0.97 or FlowJo.

### Generation of Bone Marrow Chimeric Mice

Bone marrow from donor mice (*WT*, *Stat1^–/–^*, *Stat1^α/α^, Stat1*^β/β^, *Jak1^*fl/fl*^Ncr1Cre*, and *Ly5.1*) was depleted of mature NK cells and T cells by using anti-CD49b and anti-CD5b labeled MACS beads (Miltenyi), respectively, according to the manufacturer’s instructions. *WT*, *Stat1^–/–^*, *Stat1*^α/α^, or *Stat1*^β/β^ bone marrow cells were mixed 1:1 with *Ly5.1* or *Jak1^*fl/fl*^Ncr1Cre* bone marrow cells and 4 × 10^6^ cells of the respective mix were intravenously (i.v.) injected into irradiated (9 Gy) *Ly5.1^+^* or *Jak1^*fl/fl*^Ncr1Cre* mice. Splenic NK cell maturation of donor mice (at the day of bone marrow transfer) and of chimeric mice (6 weeks after bone marrow transfer) was analyzed by flow cytometry. *Jak1^*fl/fl*^Ncr1Cre* chimeric mice were used 6 weeks after bone marrow transfer for *in vivo* cytotoxicity assays and the analysis of splenic NK cell maturation by flow cytometry.

### *In vivo* IL-15/IL-15Rα Treatment

A total of 0.5 μg rmIL-15 and 3 μg rmIL-15Rα-human-IgG1-Fc (IL-15Rα) (R&D Systems) were mixed in 200 μl PBS and incubated for 30 min at 37°C. *WT*, *Stat1^–/–^*, and *Stat1*^β/β^ mice were intraperitoneally (i.p.) injected with 200 μl IL-15/IL-15Rα complexes four times within 1 week. Two days after the last injection, splenic NK cell maturation, IFNγ production upon *ex vivo* stimulation with anti-NK1.1 antibody and *in vivo* cytotoxicity of NK cells were analyzed.

### Tumor Challenge

The murine lymphoma cell lines RMA-S (34) and RMA-Rea1 (35) were cultured in RPMI1640 medium (Sigma-Aldrich) supplemented with 10% heat-inactivated FCS (Invitrogen), L-glutamine (PAA), 50 μM 2-mercaptoethanol (Gibco), 100 U/ml penicillin and 100 μg/ml streptomycin (Sigma-Aldrich) at 37°C, 5% CO_2_ and 95% humidity. Mice were subcutaneously (s.c.) injected with 1 × 10^6^ tumor cells (RMA-S or RMA-Rea1) into shaven flanks. Ten days after injection, tumors were isolated and weighed.

### NK Cell *in vivo* Cytotoxicity Assay

A 1:1 mix (input) of major histocompatibility complex class I^low^ cells (MHC I^low^, β*2m^–/–^*) and *WT* splenocytes stained with different concentrations of carboxyfluorescein succinimidyl ester [CFSE; 5 μM (high) and 0.5 μM (low), Invitrogen] were i.v. injected into recipient mice (1 × 10^7^ cells). CFSE^high^ and CFSE^low^ cells within the spleen of the recipients (sample) were detected by flow cytometry 16 h after the injection. The cytotoxic capacity of NK cells was determined by analyzing the ratio of MHC I^low^ and *WT* cells in the injected cell population and in the spleen of recipient mice by calculating the rejection with the following formula:

$$\begin{array}{c} \%\mathrm{rejection}\mathrm{of}\\ \mathrm{MHC}I^{\mathrm{low}}\mathrm{cells} \end{array}=\left(1-\left(\frac{\begin{array}{c} \%{\mathrm{CFSE}}^{\mathrm{low}}\left(\text{input}\right)/\\ \%{\mathrm{CFSE}}^{\mathrm{high}}\left(\text{input}\right) \end{array}}{\begin{array}{c} \%{\mathrm{CFSE}}^{\mathrm{low}}\left(\text{sample}\right)/\\ \%{\mathrm{CFSE}}^{\mathrm{high}}\left(\text{sample}\right) \end{array}}\right)\right)\times 100$$

### NK Cell Stimulation *ex vivo*

Splenocyte single-cell suspensions after erythrocyte lysis were stimulated with tube-bound anti-NK1.1 (BioLegend), anti-NKp46 (Thermo Fisher Scientific) antibody or IL-12 and IL-18 (5 ng/ml and 25 ng/ml, respectively, R&D Systems and MBL) for 1 h followed by 4 h treatment with brefeldin A (Thermo Fisher Scientific) or with PMA/ionomycin (PMA/ionomycin, Cell Activation Cocktail, BioLegend) and brefeldin A for 5 h at 37°C, 5% CO_2_ and 95% humidity. Staining and flow cytometric analysis was performed as described above. For the analysis of IFNγ in the cell culture supernatant, magnetic beads purified (Miltenyi) NK cells were stimulated with IL-12 and IL-18 (5 ng/ml and 25 ng/ml, respectively, R&D Systems and MBL) or with PMA and ionomycin (50 ng/ml and 1 μg/ml, respectively, Sigma-Aldrich) for 6 and 21 h. IFNγ production was assessed using mouse IFN-gamma Quantikine ELISA (R&D Systems) according to manufacturer’s instructions.

### Statistical Analysis

GraphPad Prism^®^ version 7.00 for Mac (GraphPad Software) was used to perform one-way ANOVA or two-way ANOVA with Tukey’s *post hoc* test. R-studio IDE (36) was used to perform two-way ANOVA with Tukey’s *post hoc* test (Figure 2C). Statistical significances are depicted for each dataset.

## Results

### Absence of STAT1α Results in Impaired NK Cell Maturation

**FIGURE 1:**
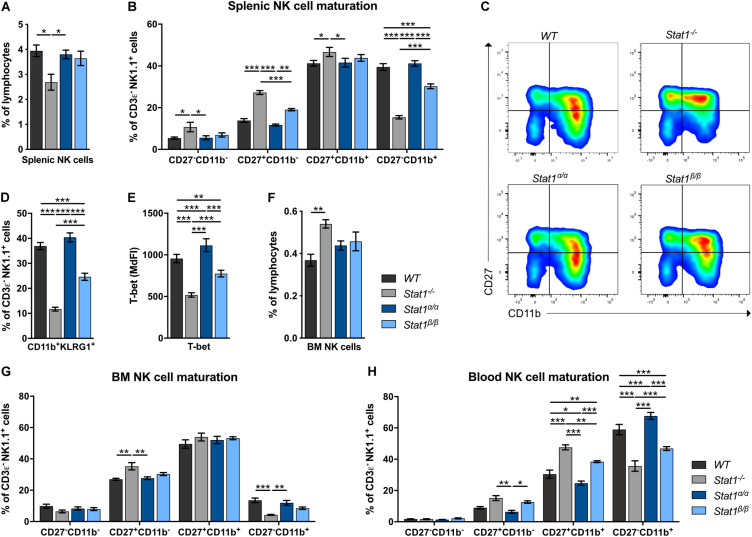
*Stat1*^β/β^, but not *Stat1*^α/α^, mice show impaired NK cell maturation. **(A–D)** The abundance of NK cells (CD3ε^–^NK1.1^+^) **(A)**, NK cell maturation subsets (CD27^–^CD11b^–^, CD27^+^CD11b^–^, CD27^+^CD11b^+^, and CD27^–^CD11b^+^) **(B,C)** and of CD11b^+^KLRG1^+^ NK cells **(D)** were analyzed in spleens of *WT*, *Stat1^–/–^*, *Stat1*^α/α^, and *Stat1*^β/β^ mice. Representative dot blots **(C)** and mean percentages ± SEM (*n* = 7–8) from three experiments **(A,B,D)** are shown. **(E)** T-bet expression was determined by intracellular staining and flow cytometry in splenic NK cells (CD3ε^–^NK1.1^+^) from *WT*, *Stat1^–/–^*, *Stat1*^α/α^, and *Stat1*^β/β^ mice. Least squares mean of median fluorescence intensities (MdFIs) ± SEM from three experiments (*n* = 6–9) are depicted. **(F,G)** The abundance of NK cells (CD3ε^–^NK1.1^+^) in the bone marrow (BM) **(F)** and NK cell maturation subsets (CD27^–^CD11b^–^, CD27^+^CD11b^–^, CD27^+^CD11b^+^, and CD27^–^CD11b^+^) in the bone marrow (BM) **(G)** and the blood **(H)** from *WT*, *Stat1^–/–^*, *Stat1*^α/α^, and *Stat1*^β/β^ mice were analyzed by flow cytometry. Mean percentages ± SEM (*n* = 3–6) from two experiments **(F–H)** are shown. *p < 0.05; **p < 0.01; ***p < 0.001.

Absence of STAT1 considerably reduces the proportion of the most mature NK cell subset in the periphery but does not affect NK cell development (9, 11). In contrast to *Stat1^–/–^* mice (10), *Stat1*^α/α^ and *Stat1*^β/β^ mice had similar frequencies of total splenic NK cells (CD3ε^–^NK1.1^+^) as *WT* mice (Figure 1A). In mice, four stages of maturation can be distinguished by the markers CD27, CD11b (Mac-1), and KLRG1 within CD3ε^–^NK1.1^+^ NK cells: progenitors (CD27^–^CD11b^–^KLRG1^–^), immature (CD27^+^CD11b^–^KLRG1^–^), M1 mature (CD27^+^CD11b^+^KLRG1^–^), and M2 mature (CD27^–^CD11b^+^KLRG1^+^) NK cells (37, 38). As in previous studies (9, 11), *Stat1^–/–^* mice showed a reduced proportion of splenic M2 mature NK cells, defined as CD27^–^CD11b^+^ (Figures 1B,C) and CD11b^+^KLRG1^+^ NK cells (Figure 1D). NK cell maturation was unaffected in *Stat1*^α/α^ mice, whereas *Stat1*^β/β^ mice had reduced frequencies of CD27^–^CD11b^+^ (Figures 1B,C) and CD11b^+^KLRG1^+^ NK cells (Figure 1D), although the NK cell maturation defect in *Stat1*^β/β^ mice was less pronounced than in *Stat1^–/–^* mice (Figures 1B–D). T-bet is a critical transcription factor to induce the transition from immature to mature NK cells (39, 40). T-bet levels in NK cells from *WT*, *Stat1^α/α^ Stat1*^β/β^, and *Stat1^–/–^* mice showed a similar pattern as the abundance of CD27^–^CD11b^+^ NK cells, except that they were slightly (*p* ≤ 0.001) higher in *Stat1*^α/α^ than in *WT* mice (Figure 1E). There are substantial differences in the tissue distribution of NK cell subsets, with the highest proportion of immature NK cells in the bone marrow (41, 42). In line with our observations in the spleen, *Stat1*^α/α^ and *Stat1*^β/β^ mice had similar frequencies of total NK cells in the bone marrow (Figure 1F) and the frequency of CD27^–^CD11b^+^ NK cells in *Stat1*^β/β^ mice was intermediate between *WT* and *Stat1^–/–^* mice (Figure 1G). A similar maturation defect was observed in blood and liver NK cells from *Stat1*^β/β^ mice (Figure 1H and Supplementary Figures 1A,B).

Taken together, these data suggest that STAT1β promotes full NK cell maturation less efficiently than STAT1α and that the absence of STAT1β does not increase NK cell maturation in the bone marrow and the periphery.

### Impaired NK Cell Maturation in the Absence of STAT1α Relates to Defects in IFNγ Signaling

**FIGURE 2:**
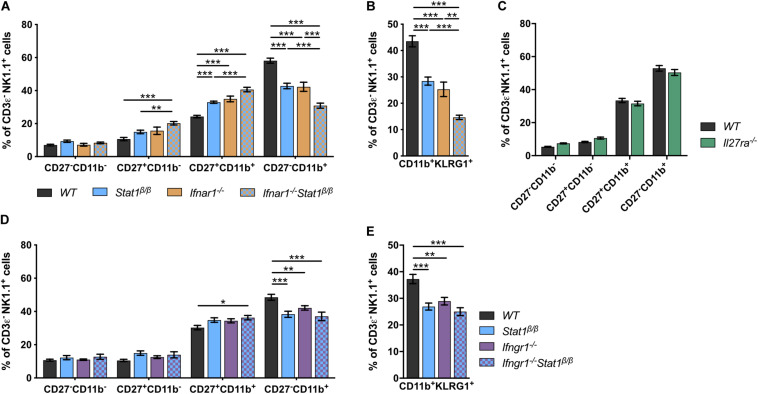
The defective NK cell maturation in *Stat1*^β/β^ mice reflects impaired IFNγ, but not IFNα/β, signaling. **(A–E)** Splenic NK cells (CD3ε^–^NK1.1^+^) from *WT, Stat1*^β/β^, *Ifnar1^–/–^*, and *Ifnar1^–/–^Stat1*^β/β^ **(A,B)**, *WT* and *Il27ra^–/–^* **(C)**, *WT, Stat1*^β/β^, *Ifngr1^–/–^*, and *Ifngr1^–/–^Stat1*^β/β^ **(D,E)** mice were analyzed for the maturation subsets CD27^–^CD11b^–^, CD27^+^CD11b^–^, CD27^+^CD11b^+^, and CD27^–^CD11b^+^ **(A,C,D)** and CD11b^+^KLRG1^+^ **(B,E)** by flow cytometry. Mean percentages ± SEM from eight (*n* = 5–18) **(A,B)**, two (*n* = 12) **(C)** and six experiments (*n* = 10–14) **(D,E)** are depicted. *p < 0.05; **p < 0.01; ***p < 0.001.

Signal transducer and activator of transcription 1 is best known for its central role in signaling by all types of IFNs but also contributes to signaling by other cytokines, such as IL-21, IL-27, and IL-35 (43–45). Mice lacking the type I IFN receptor chain 1 (*Ifnar1^–/–^*) or the IFNAR1-associated JAK TYK2 (*Tyk2^–/–^*) have NK cell maturation defects comparable to those of *Stat1*^β/β^ mice (46–49). To determine whether the defect in *Stat1*^β/β^ mice is due to alterations of type I IFN responses, we generated *Ifnar1^–/–^Stat1*^β/β^ double mutant mice and comparatively analyzed NK cell maturation in *WT*, *Ifnar1^–/–^*, *Stat1*^β/β^, and *Ifnar1^–/–^Stat1*^β/β^ mice. The frequency of splenic NK cells did not differ between *WT*, *Ifnar1^–/–^*, *Stat1*^β/β^, and *Ifnar1^–/–^Stat1*^β/β^ mice (Supplementary Figure 2A). *Stat1*^β/β^ and *Ifnar1^–/–^* mice showed similar NK cell maturation profiles, whereas *Ifnar1^–/–^Stat1*^β/β^ double mutant mice showed a considerably lower frequency of CD27^–^CD11b^+^ and CD11b^+^KLRG1^+^ NK cells than single mutant mice (Figures 2A,B). This additive effect indicates that STAT1α also drives NK cell maturation independently of IFNAR1. We next analyzed NK cell maturation in mice that are unresponsive to IL-27 (*Il27ra^–/–^*) or IFNγ (*Ifngr1^–/–^*). The relative abundance of the four NK cell maturation stages did not differ between *Il27ra^–/–^* and *WT* mice (Figure 2C), suggesting that IL-27 signaling is not required for splenic NK cell maturation. However, the frequency of total NK cells was slightly reduced in *Il27ra^–/–^* mice compared to *WT* mice (Supplementary Figure 2B). *Ifngr1^–/–^* mice had a lower frequency of CD27^–^CD11b^+^ and CD11b^+^KLRG1^+^ NK cells than *WT* mice (Figures 2D,E). NK cells from *Ifngr1^–/–^Stat1*^β/β^ double mutant mice had a similar frequency of all four NK cell maturation stages and of CD11b^+^KLRG1^+^ NK cells as *Ifngr1^–/–^* and *Stat1*^β/β^ single mutant mice, suggesting that IFNγ and STAT1α promote NK cell maturation through a common mechanism. The total NK cell frequency did not differ between *Ifngr1^–/–^*, *Ifngr1^–/–^Stat1*^β/β^, *Stat1*^β/β^, and *WT* mice (Supplementary Figure 2C).

Taken together these data suggest that in addition to type I IFN (IFNα/β), type II IFN (IFNγ) signaling contributes to splenic NK cell maturation under homeostatic conditions and that the full activity of the latter depends on the presence of the STAT1α isoform. Moreover, we show that IL-27 signaling is not required for NK cell maturation but might affect survival, proliferation or recruitment of splenic NK cells.

### STAT1α Is Required for Full NK Cell Functionality and Tumor Surveillance

**FIGURE 3:**
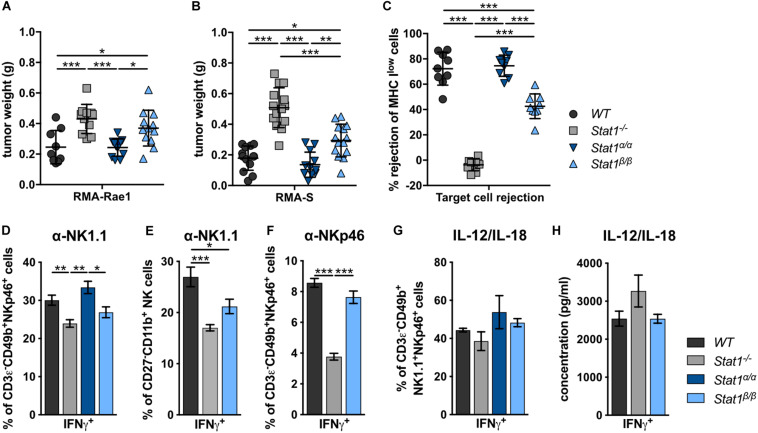
*Stat1*^β/β^, but not *Stat1*^α/α^, mice show reduced NK cell-dependent anti-tumor activity, *in vivo* cytotoxicity and impaired production of IFNγ in response to actR stimulation. **(A,B)** 1 × 10^6^ RMA-Rae1 **(A)** or RMA-S **(B)** cells were s.c. injected in both flanks of *WT*, *Stat1^–/–^*, *Stat1*^α/α^, and *Stat1*^β/β^ mice. Ten days after injection the tumors were isolated and weighed. Tumor weight (*n* = 9–14) and mean values ± SD from two experiments are shown. **(C)** *WT*, *Stat1^–/–^*, *Stat1*^α/α^, and *Stat1*^β/β^ mice were i.v. injected with a 1:1 mix of CFSE^low^ stained *WT* and CFSE^high^ stained MHC I^low^ (β*2m^–/–^*) splenocytes. The ratio of CFSE^low^ and CFSE^high^ cells in the spleen of recipient mice was determined 16 h after injection by flow cytometry. Mean percentages ± SD (*n* = 9) from three experiments are given. **(D–F)** Splenocytes from *WT*, *Stat1^–/–^*, *Stat1*^α/α^, and *Stat1*^β/β^ mice were stimulated with tube-bound anti-NK1.1 antibody **(D,E)**, tube-bound anti-NKp46 antibody **(F)** and IL-12 (5 ng/ml) and IL-18 (25 ng/ml) **(G)**, and incubated in the presence of brefeldin A. IFNγ production was analyzed by intracellular staining of NK cells **(D,F,G)** or M2 mature NK cells (CD27^–^CD11b^+^) **(E)**. Mean percentages ± SEM from six (*n* = 6–18) **(D)**, three experiments (*n* = 9) **(E)**, one experiment (*n* = 3) **(F)**, and two experiments (*n* = 5–6) **(G)** are shown. **(H)** Magnetic beads-purified NK cells were stimulated with IL-12 (5 ng/ml) and IL-18 (25 ng/ml) for 6 h. IFNγ was determined in the culture supernatant by ELISA. Mean IFNγ concentrations ± SEM from one experiment (*n* = 3) is depicted. *p < 0.05; **p < 0.01; ***p < 0.001.

To investigate the impact of STAT1 isoforms on NK cell functions, we analyzed NK cell-dependent tumor growth control using cell lines that either express an activating receptor ligand (RMA-Rae1) or lack inhibitory receptor ligands (RMA-S). RMA-Rae1 tumors grew significantly bigger in *Stat1^–/–^* and *Stat1*^β/β^ mice compared to *Stat1*^α/α^ and *WT* mice (Figure 3A). A similar pattern of tumor growth was seen upon injection of RMA-S cells, which grew to significantly bigger tumors in *Stat1^–/–^* and *Stat1*^β/β^ mice compared to *Stat1*^α/α^ and *WT* mice, albeit tumors were smaller in *Stat1*^β/β^ than in *Stat1^–/–^* mice (Figure 3B). Impaired NK cell functionality in *Stat1*^β/β^ mice was further supported by *in vivo* cytotoxicity assays. As expected, *Stat1^–/–^* mice were not able to reject major histocompatibility complex class I (MHC class I) low-expressing NK target cells (MHC I^low^ = β*2m^–/–^* cells, Figure 3C). MHC I^low^ target cell rejection in *Stat1*^β/β^ mice was intermediate between *WT* and *Stat1^–/–^* mice, whereas *Stat1*^α/α^ mice rejected target cells as efficiently as *WT* mice (Figure 3C). Together with the data from the tumor transplants, this indicates that *Stat1*^β/β^ mice have a better missing-self recognition than *Stat1^–/–^* mice, whereas NKG2D-dependent killing is similarly impaired in *Stat1*^β/β^ and *Stat1^–/–^* mice.

In addition to impaired NK cell cytotoxicity and NK cell-dependent tumor surveillance, NK cells from *Stat1^–/–^* mice show reduced production of IFNγ in response to stimulation of the NK cell activating receptors (actRs) NK1.1 and NKp46 (Figures 3D–F). IFNγ production by *Stat1*^β/β^ NK cells in response to stimulation with anti-NK1.1 antibody was intermediate between *WT* and *Stat1^–/–^* NK cells, albeit the difference in IFNγ production was only statistically significant (*p* < 0.05) for the comparison of *Stat1*^β/β^ with *Stat1*^α/α^ but not with *WT* NK cells (Figure 3D). The most mature splenic NK cell population (CD27^–^CD11b^+^) shows the highest IFNγ production in response to activation of the actR NKp46 (50). To test whether the decrease in anti-NK1.1 antibody-induced IFNγ production of NK cells from *Stat1*^β/β^ mice is due to the reduction in this NK cell subset, we gated for CD27^–^CD11b^+^ NK cells and analyzed the frequency of IFNγ-positive cells within this population. Similar to our observation with the total NK cell pool, fewer CD27^–^CD11b^+^ NK cells from *Stat1^–/–^* and *Stat1*^β/β^ mice produced IFNγ in response to NK1.1 stimulation than *WT* cells (Figure 3E). To test whether this is also observed upon activation of another actR, we stimulated *WT*, *Stat1^–/–^* and *Stat1*^β/β^ NK cells with anti-NKp46 antibody. Similar to NK1.1 activation, we observed a modestly reduced frequency of *Stat1*^β/β^ NK cells producing IFNγ compared to their *WT* counterparts, although this did not reach statistical significance with the threshold set at *p* < 0.05 (Figure 3F). In contrast to NK1.1 and NKp46 stimulation, NK cells from mice of all four genotypes showed unimpaired IFNγ production in response to IL-12/IL-18 (Figures 3G,H and Supplementary Figure 3D) or PMA/ionomycin (Supplementary Figures 3A–C).

Taken together, these data show that the presence of STAT1α in NK cells and/or accessory cells is required for full-fledged NK cell-mediated tumor growth control and target cell rejection, modestly increases IFNγ production in response to NK1.1 and NKp46 activation and does not impact on IFNγ production in response to IL-12/IL-18 or PMA/ionomycin. Moreover, the data show that STAT1β is dispensable for NK cell functionality.

### STAT1α in the Environment Rather Than in NK Cells Themselves Promotes NK Cell Maturation

**FIGURE 4:**
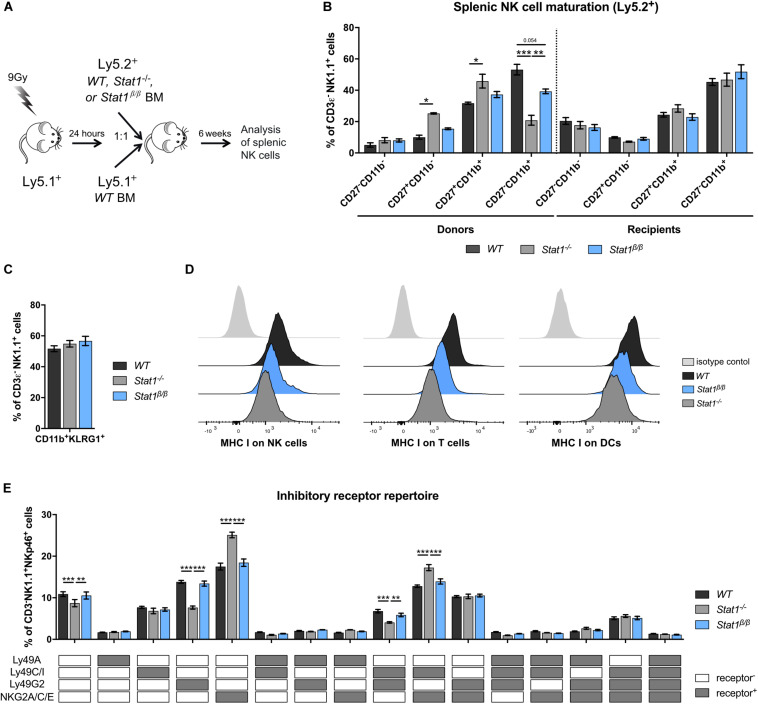
*Stat1*^β/β^ NK cells exhibit normal maturation in a *WT* environment and *Stat1*^β/β^ mice have reduced MHC class I surface levels on various splenocyte types but an unchanged NK cell inhibitory receptor repertoire. **(A)** Scheme illustrating the mixed bone marrow chimera experiments. **(B,C)** Splenocytes from donor and bone marrow chimeric mice (Ly5.2^+^) were analyzed for the NK cell (CD3ε^–^NK1.1^+^) maturation subsets CD27^–^CD11b^–^, CD27^+^CD11b^–^, CD27^+^CD11b^+^, and CD27^–^CD11b^+^ **(B)** and for CD11b^+^KLRG1^+^ cells **(C)**. **(D)** Surface levels of MHC class I molecules (MHC I) on splenic NK cells (left), T cells (middle) and DCs (right) from *WT*, *Stat1^–/–^* and *Stat1*^β/β^ mice were determined by flow cytometry. Histograms show one representative sample per cell type and genotype and the isotype control, which was comparable for cells from mice of all genotypes. **(E)** The inhibitory receptor repertoire of splenic NK cells (CD3ε^–^NK1.1^+^) from *WT*, *Stat1^–/–^*, and *Stat1*^β/β^ mice was determined by flow cytometry. NK subsets were defined by combinatorial expression of Ly49A, Ly49C/I, Ly49G2, and NKG2A, as determined by flow cytometry, and analyzed by Boolean gating. White and gray boxes indicate whether the population is positive (gray) or negative (white) for the inhibitory receptors listed. Mean percentages ± SEM from three (*n* = 5–8) **(B,C)** and two (*n* = 5–6) **(E)** experiments are shown. *p < 0.05; **p < 0.01; ***p < 0.001.

Having established that the presence of STAT1α, but not STAT1β, is important for NK cell functionality, we focused our further analysis on mice lacking STAT1α (*Stat1*^β/β^ mice). Previous studies using bone marrow chimera experiments have demonstrated that STAT1 in the environment rather than in NK cells themselves is required for the upregulation of the maturation markers CD11b and KLRG1 (11). These findings would imply that NK cells from *Stat1*^β/β^ mice mature normally when transferred into a *WT* environment. To test this hypothesis, we generated mixed bone marrow chimeric mice by injecting a 1:1 mix of *WT* (*Ly5.1^+^*) with *WT* (*Ly5.2^+^*), *WT* (*Ly5.1^+^*) with *Stat1^–/–^* (*Ly5.2^+^*) or *WT* (*Ly5.1^+^*) with *Stat1*^β/β^ (*Ly5.2^+^*) bone marrow cells into lethally irradiated *WT* mice (*Ly5.1^+^*). Splenic NK cell maturation of Ly5.2^+^ cells was analyzed before (donors) and 6 weeks after transplantation (recipients) (Figure 4A). Splenic NK cells from *Stat1^–/–^* and *Stat1*^β/β^ donor mice showed impaired maturation, whereas *Stat1^–/–^* and *Stat1*^β/β^ NK cells that developed in a *WT* environment were indistinguishable from *WT* NK cells with respect to the frequency of CD27^–^CD11b^+^ (Figure 4B) and CD11b^+^KLRG1^+^ NK cells (Figure 4C). Thus, cell-extrinsic STAT1α promotes NK cell maturation in the spleen.

### Absence of STAT1α Results in Reduced MHC Class I Levels on Splenocytes but Does Not Impact on the Inhibitory Receptor Repertoire of Splenic NK Cells

MHC class I molecules are ligands for inhibitory NK cell receptors and are tightly linked to NK cell education, which is required for NK cells to acquire full responsiveness to actR stimulation and cytotoxic capacity (51–53). IFNγ induces MHC class I genes through STAT1 (54) and a number of studies have highlighted the importance of STAT1 for MHC class I expression *in vivo* (11, 55, 56). To investigate whether the absence of STAT1α alters MHC class I levels, we determined MHC class I surface levels on *Stat1*^β/β^ compared to *WT* and *Stat1^–/–^* splenocytes. As expected, MHC class I surface levels were strongly reduced on *Stat1^–/–^* splenocytes, including NK cells, T cells, dendritic cells (DCs) (Figure 4D and Supplementary Figure 4A), macrophages and B cells (Supplementary Figures 4B,C). All splenocytes analyzed from *Stat1*^β/β^ mice showed intermediate MHC class I levels between *WT* and *Stat1^–/–^* cells, although the extent of reduction compared to *WT* cells varied among cell types (Figure 4D and Supplementary Figures 4A–C).

Apart from functional read-outs, the NK cell inhibitory receptor repertoire is used as a measure for NK cell education. Several NK cell subsets have been defined by the expression of different combinations of the inhibitory receptors Ly49A, Ly49C/I, Ly49G2, and NKG2A (57, 58). *Stat1^–/–^* mice had altered frequencies of five of these subsets compared to *WT* mice, including an increased abundance of NKG2A/C/E^+^ and NKG2A/C/E^+^Ly49C/I^+^ NK cells and a reduced abundance of Ly49G2^+^ and Ly49G2^+^Ly49C/I^+^ NK cells (Figure 4E). In contrast, we did not observe differences in the frequency of any of these NK cell subsets between *WT* and *Stat1*^β/β^ mice (Figure 4E). In addition, levels of Ly49A, Ly49C/I, and NKG2A/C/E did not differ between *WT* and *Stat1*^β/β^ mice on the total NK cell pool, whereas levels of Ly49G2 were modestly increased on NK cells from *Stat1^–/–^* and *Stat1*^β/β^ compared to *WT* mice (Supplementary Figures 4D–G).

Natural killer cell activity is tightly controlled by the balance between activating and inhibitory receptors (59). Except for a modest reduction of the actR NKG2D on NK cells in *Stat1*^β/β^ compared to *WT* mice, levels of NK1.1, NKp46 and Ly49D did not differ between NK cells from *Stat1*^β/β^ and *WT* mice (Supplementary Figures 4H–K).

Taken together these data show that the absence of STAT1α results in reduced MHC class I levels on several cell types in the spleen but, in contrast to the total absence of STAT1, does not impact the NK cell inhibitory receptor repertoire, suggesting that STAT1β is capable of upregulating MHC class I molecules to levels that are sufficient to support NK cell education. Moreover, these data show that STAT1β is capable of upregulating the actRs Ly49D and NKG2D, albeit the latter slightly less efficiently than STAT1α, whereas NK1.1 and NKp46 are regulated independent of STAT1 and its isoforms.

### Absence of STAT1α or IFNGR1 Results in Reduced Surface Levels of IL-15Rα in Splenic DCs, Monocytes and Macrophages

**FIGURE 5:**
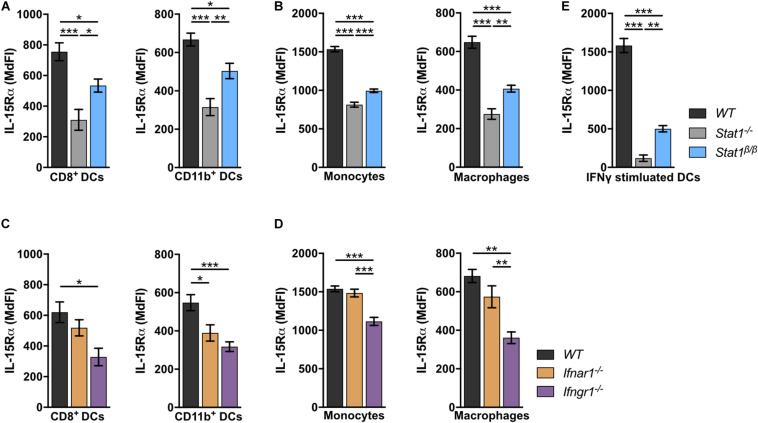
*Stat1^–/–^*, *Stat1^β/β^, Ifnar1^–/–^*, and *Ifngr1^–/–^* mice have reduced IL-15Rα surface levels on various splenocytes. **(A–D)** Surface levels of IL-15Rα were determined on splenic DCs subsets **(A,C)**, monocytes and macrophages **(B,D)** from *WT*, *Stat1^–/–^*, and *Stat1*^β/β^ mice **(A,B)** and from *WT*, *Ifnar1^–/–^*, and *Ifngr1^–/–^* mice **(C,D)** using flow cytometry. Mean MdFIs ± SEM (*n* = 7–11) from three experiments are shown **(A–D)**. **(E)** Magnetic beads-isolated, splenic DCs from *WT*, *Stat1^–/–^*, and *Stat1*^β/β^ mice were treated with or without IFNγ (100 U/ml) after 16 h and IL-15Rα surface levels were determined by flow cytometry. Mean MdFIs (IFNγ treated minus untreated control) ± SEM (*n* = 4) from two experiments are shown. *p < 0.05; **p < 0.01; ***p < 0.001.

IL-15 is an important cell-extrinsic signal for NK cell homeostasis (60, 61). It is chaperoned to the cell surface by the co-expressed IL-15 receptor α (IL-15Rα) and trans-presented to IL-15Rβ-expressing cells, such as NK cells (62–67). Under homeostatic conditions, IL-15/IL-15Rα trans-presentation to NK cells is mediated by DCs and monocytes (50, 68). To investigate whether the absence of STAT1α affects IL-15 trans-presentation, we analyzed IL-15Rα surface levels on *WT*, *Stat1^–/–^*, and *Stat1*^β/β^ splenocytes. In *Stat1^–/–^* mice, IL-15Rα surface levels on CD8^+^ DCs and CD11b^+^ DCs (Figure 5A), monocytes and macrophages (Figure 5B) were profoundly reduced compared to IL-15Rα surface levels on *WT* cells. Again, *Stat1*^β/β^ cells showed an intermediate phenotype between *Stat1^–/–^* and *WT* cells (Figures 5A,B). As STAT1 regulates its own transcription (69), we also analyzed STAT1 protein levels in these cells using an antibody that recognizes both STAT1 isoforms. In contrast to our findings in spleen and liver homogenates and in bone marrow-derived macrophages (16), splenic CD8^+^ DCs, CD11b^+^ DCs, monocytes and macrophages from *Stat1*^β/β^ mice showed reduced levels of STAT1 compared to *WT* cells (Supplementary Figures 5A–C). IL-15 and IL-15Rα are induced by IFNα/β and IFNγ (70–73). Previous studies have shown that in response to immune challenge, type I IFN upregulates IL-15/IL-15Rα expression on bone marrow-derived DCs (74). As it remained unclear how IL-15/IL-15Rα expression is regulated under homeostatic conditions, we analyzed IL-15Rα levels on splenic DCs, monocytes and macrophages from *Ifnar1^–/–^* and *Ifngr1^–/–^* mice. IL-15Rα surface levels were clearly reduced in CD11b^+^ DCs, only slightly (*p* > 0.05) in CD8^+^ DCs and macrophages but not in monocytes from *Ifnar1^–/–^* mice (Figures 5C,D). In contrast, *Ifngr1^–/–^* mice had considerably reduced IL-15Rα levels in all these cell types (Figures 5C,D). To confirm that IFNγ is capable of upregulating IL-15Rα and that this is less efficient in the absence of STAT1α, we treated splenic DCs overnight with IFNγ. As expected, upon IFNγ treatment DCs from *Stat1*^β/β^ mice had lower IL-15Rα levels than IFNγ treated *WT* DCs but higher than IFNγ treated *Stat1^–/–^* DCs (Figure 5E).

Collectively, these data show that under homeostatic conditions IFNγ signaling and STAT1α augment IL-15Rα surface levels on DCs, monocytes and macrophages, whereas type I IFNs specifically increase IL-15Rα on CD11b^+^ DCs.

### NK Cell Maturation Can Be Rescued by Treatment of *Stat1*^β/β^ Mice With Exogenous IL-15/IL-15Rα

**FIGURE 6:**
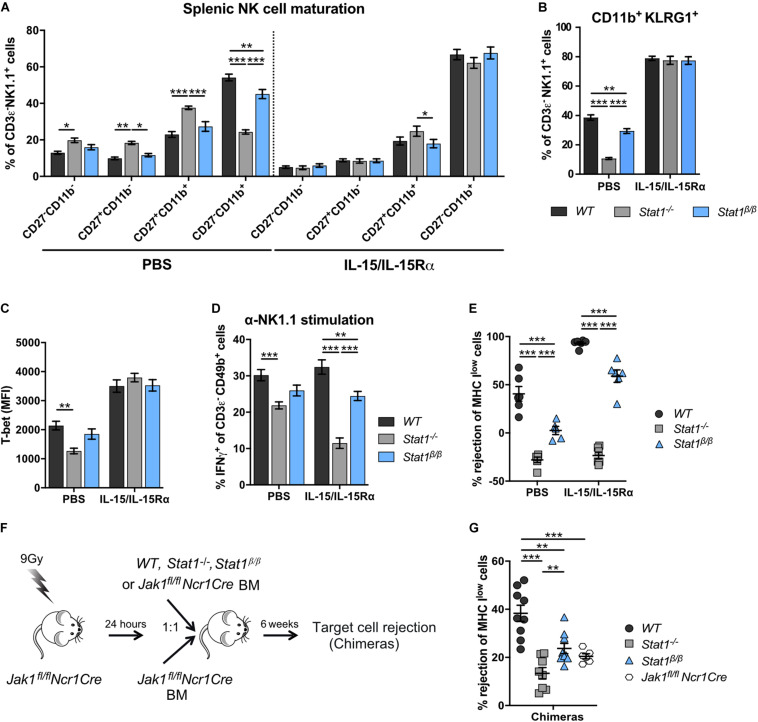
Treatment with exogenous IL-15/IL-15Rα complexes rescues NK cell maturation but not functionality in *Stat1^–/–^* and *Stat1*^β/β^ mice and development of NK cells from *Stat1^–/–^* and *Stat1*^β/β^ mice in a STAT1-proficient environment does not rescue *in vivo* NK cell cytotoxicity. **(A–E)** *WT*, *Stat1^–/–^*, and *Stat1*^β/β^ mice were treated with PBS or with IL-15/IL-15Rα for 1 week. Splenic NK cells (CD3ε^–^ NK1.1^+^) of these mice were analyzed for the maturation subsets CD27^–^CD11b^–^, CD27^+^CD11b^–^, CD27^+^CD11b^+^, and CD27^–^CD11b^+^ **(A)**, CD11b^+^KLRG1^+^ **(B)**, and intracellular T-bet levels **(C)**. Mean percentages ± SEM (*n* = 11–12) from four experiments **(A,B)** and mean MdFIs ± SEM (*n* = 6) from two experiments **(C)** are shown. Splenocytes from PBS or IL-15/IL-15Rα treated mice were analyzed for IFNγ production in response to NK1.1 stimulation as described in the legend to Figure 3D **(D)** and *in vivo* cytotoxicity assay was performed **(E)**. Mean percentages ± SEM from three (*n* = 9) **(D)** and two (*n* = 6) **(E)** experiments are shown. For simplicity, statistical significances are only indicated for genotype comparisons **(A–E)**. **(F)** Scheme illustrating mixed bone morrow chimera experiments. **(G)** Bone marrow chimeric mice (chimeras) and *Jak1^*fl/fl*^Ncr1Cre* controls were used for *in vivo* NK cell cytotoxicity assays using MHC I^low^ target cells as described in the legend to Figure 3C. Mean percentages ± SEM (*n* = 6–10) from three experiments are shown **(G)**. *p < 0.05; **p < 0.01; ***p < 0.001.

We next tested whether IL-15/IL-15Rα complex treatment restores NK cell maturation in *Stat1^–/–^* and *Stat1*^β/β^ mice. Sustained IL-15/IL-15Rα complex treatment of *Stat1^–/–^* and *Stat1*^β/β^ mice resulted in similar frequencies of CD27^–^CD11b^+^ and CD11b^+^KLRG1^+^ NK cells as in *WT* mice (Figures 6A,B). PBS treatment was used as control and did not impact on the frequencies of CD27^–^CD11b^+^ and NK1.1^+^KLRG1^+^ NK cells (Figures 6A,B). In addition, IL-15/IL-15Rα treatment of *Stat1^–/–^* and *Stat1*^β/β^ mice restored T-bet levels to levels observed in NK cells from *WT* mice (Figure 6C). It is important to note, that the expected increase in spleen weight and cellularity and total NK cell numbers by the sustained IL-15/IL-15Rα treatment was not different between mice of the three genotypes (**Supplementary Figures 6A–C**).

### NK Cell-Intrinsic STAT1α Is Required for Proper NK Cell Functionality

The positive effect of IL-15/IL-15Rα complex treatment on NK cell maturation prompted us to investigate the treatment’s impact on NK cell effector functions. In PBS-treated groups, IFNγ production in response to anti-NK1.1 antibody was intermediate in NK cells from *Stat1*^β/β^ mice between NK cells from *WT* and *Stat1^–/–^* mice (Figure 6D), which is in line with data from naïve mice (Figure 3D). IL-15/IL-15Rα complex treatment even decreased IFNγ production by *Stat1^–/–^* NK cells and did not rescue the defect of *Stat1*^β/β^ NK cells (Figure 6D). Similar results were observed for *in vivo* NK cell cytotoxicity. Treatment with IL-15/IL-15Rα increased the rejection of target cells in *WT* and *Stat1*^β/β^ mice compared to the respective PBS-treated groups, demonstrating that IL-15/IL-15Rα treatment can increase the cytotoxicity in mice of both genotypes (Figure 6E). However, the level of rejection was still lower in IL-15/IL-15Rα-treated *Stat1*^β/β^ than in IL-15/IL-15Rα-treated *WT* mice, indicating that NK cell-mediated killing cannot be fully rescued by treatment of *Stat1*^β/β^ with IL-15/IL-15Rα (Figure 6E).

Having established that IL-15/IL-15Rα treatment fully restores NK cell maturation but not NK cell effector functions in *Stat1*^β/β^ mice, we next tested whether NK cell functionality can be restored when NK cells mature in a STAT1-proficient environment. To enable functional assays *in vivo*, we made use of *Jak1^*fl/fl*^Ncr1Cre* mice, which have strongly reduced NK cell numbers (32). We generated bone marrow chimeras by injecting *WT*, *Stat1^–/–^*, or *Stat1*^β/β^ mixed with *Jak1^*fl/fl*^Ncr1Cre* bone marrow cells or only *Jak1^*fl/fl*^Ncr1Cre* bone marrow cells into irradiated *Jak1^*fl/fl*^Ncr1Cre* mice and tested NK cell cytotoxicity using MHC I^low^ target cells (Figure 6F). *Jak1^*fl*/fl^Ncr1Cre* mice are not completely devoid of NK cells and were still able to reject MHC I^low^ target cells upon transfer of *Jak1^*fl*/fl^Ncr1Cre* bone marrow cells, albeit considerably less efficiently than *Jak1^*fl*/fl^Ncr1Cre* mice that received *WT* bone marrow cells (Figure 6G). In contrast to *WT* cells, *Stat1^–/–^* or *Stat1*^β/β^ bone marrow cells could not restore NK cell cytotoxicity in mixed bone marrow chimeras (Figure 6G). The total abundance of NK cells in recipient *Jak1^*fl/fl*^Ncr1Cre* mice did not differ between mice that received *WT*, *Stat1^–/–^*, or *Stat1*^β/β^ bone marrow cells, although it did not reach the level of *WT* mice (Supplementary Figure 6D, compare to Figure 1A). As expected, the maturation of *Stat1^–/–^* and *Stat1*^β/β^ NK cells was rescued when they matured in *Jak1^*fl/fl*^Ncr1Cre* mice (Supplementary Figures 6E,F). Thus, transfer into a STAT1-proficient environment rescues terminal maturation but not cytotoxic capacity of *Stat1^–/–^* and *Stat1*^β/β^ NK cells.

As the data indicate NK cell-intrinsic functions of STAT1, we next analyzed STAT1 protein levels in *WT*, *Stat1*^α/α^ and *Stat1*^β/β^ NK cells. *Stat1^–/–^* NK cells were included as controls. *WT* NK cells express predominantly STAT1α and STAT1 protein levels did not grossly differ in the total NK cell pool from *Stat1*^α/α^ and *Stat1*^β/β^ mice as determined by western blot (Supplementary Figure 6G). STAT1 levels in NK cell subsets could only be analyzed by flow cytometry and an antibody that recognizes both STAT1 isoforms. Interestingly, STAT1 protein levels differed in NK cell subsets from *WT* mice, with the highest expression in CD27^+^CD11b^–^ NK cells. All NK cell subsets from *Stat1*^β/β^ mice showed reduced levels of STAT1 compared to NK cells from *WT* mice, whereas NK cells from *Stat1*^α/α^ mice showed similar STAT1 levels as *WT* NK cells, except for a modest increase of STAT1 in the CD27^+^CD11b^–^ subset (Supplementary Figure 6H).

Collectively, these data show that NK cell maturation but not NK cell effector functions can be restored by exogenous IL-15/IL-15Rα treatment of *Stat1^–/–^* and *Stat1*^β/β^ mice and that NK cell cytotoxicity is driven by NK cell-intrinsic functions of STAT1. Moreover, data indicate a differential regulation of total STAT1 expression in NK cell subsets and a requirement for STAT1α for the upregulation or maintenance of total STAT1 levels in all NK cell subsets.

## Discussion

In this study, we have used gene-targeted mice that express either only STAT1α (*Stat1*^α/α^) or only STAT1β (*Stat1*^β/β^) to assess the function of the individual STAT1 isoforms in primary NK cells and *in vivo*. We are the first to show that the two splice isoforms of STAT1 differ in regard to their ability to promote NK cell maturation, cytotoxicity and NK cell-dependent tumor immune surveillance.

Our data show that STAT1β drives NK cell maturation less efficiently than STAT1α but suffices to provide NK cells with WT levels of the inhibitory receptor repertoire. We uncovered a novel role of the IFNγ signaling cascade in the regulation of NK cell maturation under homeostatic conditions. Terminal NK cell maturation depends on the presence of functional type I IFN signaling, as evidenced by the reduced abundance of the most mature NK cells in *Ifnar1^–/–^*, *Tyk2^–/–^*, and *Stat1^–/–^* mice (9, 11, 46–49, 75). In contrast to our data, a previous study employing *Ifng^–/–^* mice did not find evidence for an involvement of IFNγ in NK cell maturation (76). Our finding that NK cells from *Stat1^β/β^ Ifnar1^–/–^* double*-*mutant mice have a more pronounced maturation defect than NK cells from the single mutant mice, clearly indicates that STAT1 promotes NK cell maturation also independent of IFNAR1. Moreover, *Stat1*^β/β^ and *Ifngr1^–/–^* single and double mutant mice have a superimposable NK cell maturation defect, supporting the hypothesis that STAT1 functions downstream of IFNGR1 promote NK cell maturation. This is also in line with functional differences of STAT1 isoforms in IFNγ- but not IFNβ-mediated responses in primary macrophages and *in vivo* (16).

Using mixed bone marrow chimera experiments we show that STAT1α in the environment rather than in NK cells themselves is required for full NK cell maturation in the spleen, which is consistent with findings in mice lacking both STAT1 isoforms (11). It is well established that IL-15 trans-presentation via the IL-15Rα on DCs, macrophages and monocytes is important for NK cell homeostasis and terminal maturation in a dose-dependent manner (50, 68, 77). We report that STAT1β is less efficient than STAT1α in upregulating IL-15Rα on DCs, macrophages and monocytes and that this correlates with differences of STAT1 isoforms in promoting NK cell maturation. Type I IFNs regulate IL-15 trans-presentation and NK cell maturation upon pathogen challenge (74). We show that IFNγ is of major importance for the regulation of IL-15Rα on macrophages, monocytes and DCs under homeostatic conditions, whereas type I IFNs affect homeostatic IL-15Rα levels specifically on DCs. The latter is supported by our previous findings that the absence of the IFNAR1-associated TYK2 reduces IL-15Rα levels on DCs but not macrophages and monocytes (49). IL-15Rα and IL-15 are transcriptionally regulated by the STAT1 target gene interferon regulatory factor 1 (IRF1), which cell-extrinsically promotes functional NK cell development (73, 78–80). STAT1β has a strong defect in the upregulation of IRF1 in response to IFNγ (15, 16), prompting the speculation that the impaired NK cell maturation in the absence of STAT1α reflects an impaired IFNγ-IRF1 axis in IL-15 trans-presenting cells. However, it is also possible that the failure to induce IL-15Rα in the absence of STAT1α reflects reduced total STAT1 protein levels in trans-presenting cells and a consequent decrease in IFNγ and type I IFN signaling. Further experiments will be required to distinguish between these possibilities.

Natural killer cells acquire functional capacity through the interaction of inhibitory receptors with MHC class I molecules, a process that is referred to as NK cell education (81, 82). We and others have observed reduced levels of MHC class I molecules on the surface of various cell types in the absence of STAT1 (55, 56). Absence of STAT1α reduced MHC class I surface levels on all splenocytes analyzed, including DCs, macrophages, NK cells, T cells, and B cells, albeit to a lesser degree than the absence of both STAT1 isoforms. However, in contrast to the complete lack of STAT1, absence of STAT1α did not result in defects in NK cell education with respect to the inhibitory receptor repertoire. MHC class I deficiency also reduces the frequency of KLRG1^+^ NK cells (11, 83, 84) and modestly affects surface levels of CD27 and CD11b (84, 85). It is thus possible that the impaired upregulation of MHC class I molecules by STAT1β contributes to the NK cell maturation defect in STAT1α-deficient mice.

IL-15/IL-15Rα complex treatment of mice only expressing STAT1β restored splenic NK cell maturation but not NK cell functionality, as assessed by their ability to produce IFNγ in response to NK1.1 stimulation and to reject NK target cells *in vivo*. Similarly, NK cell maturation but not functionality could be restored by transfer of *Stat1*^β/β^ bone marrow into a STAT1-proficient environment (i.e., mixed bone marrow chimeras using *Jak1^*fl/fl*^Ncr1Cre* recipient mice). This suggests that the functional defects in *Stat1*^β/β^ mice are not solely due to impaired NK cell maturation and that NK cell-intrinsic STAT1α is required for full NK cell cytotoxicity. NK cells from *Stat1*^β/β^ mice have lower levels of total STAT1 than NK cells from *WT* or *Stat1*^α/α^ mice. Thus, it remains elusive whether STAT1α promotes NK cell cytotoxicity by increasing total *Stat1* expression or other mechanisms that depend on its C-terminal TAD, which is missing in STAT1β. NK cell-intrinsic functions of STAT1 have also been reported to increase IFNγ and granzyme B production upon vaccinia virus infection in co-culture and adoptive transfer experiments (12). NK cell-intrinsic functions of STAT1 are also supported by the finding that STAT1 is recruited to the site of target cell interaction in NK cells (10). Moreover, a recent study employing transposase accessible chromatin with sequencing (ATAC-seq) indicated an involvement of STAT1 in the transcription factor network that regulates NK cell differentiation (86). The STAT1 C-terminal TAD includes serine 727 (S727), whose phosphorylation positively and negatively affects the transcriptional activity of STAT1 (87, 88). Mutation of S727 to alanine (STAT1-S727A) has no impact on NK cell maturation but increases NK cell cytotoxicity and NK cell-dependent tumor growth control (9). Together with our data, this indicates that the C-terminal TAD of STAT1 has a dual role in NK cells: it is required for full-fledged STAT1 expression and anti-tumor activity but it is also targeted by negative regulatory circuits that control NK cell cytotoxicity through its S727 phosphorylation.

Another interesting finding of our study is that absence of STAT1β does not affect NK cell maturation, cytotoxicity and anti-tumor activity. This is in line with our findings in primary macrophages and the innate immune defense against bacterial and viral infections *in vivo* (16) and raises the question about the physiological relevance of the STAT1β isoform. Overexpression studies indicated dominant negative and positive regulatory effects of STAT1β on STAT1α in B cell lines and carcinoma cells, respectively (26, 28). It seems possible that STAT1β needs to reach a specific threshold level to impact STAT1α functions and that the effects may depend on the cellular context. However, it is currently unclear how STAT1 splicing is regulated. The relatively low STAT1β levels in *WT* NK cells may explain that its absence does not affect NK cell functionality. However, future experiments are required to test this possibility and to investigate whether STAT1β levels increase in NK cells or accessory cells under pathological conditions.

## Conclusion

We provide evidence that STAT1β is less efficient in driving NK cell maturation, cytotoxicity and NK cell-dependent tumor surveillance than STAT1α. Moreover, our study supports the concept that NK cell maturation is mainly driven by cell-extrinsic mechanisms, whereas cytotoxicity is also promoted by cell-intrinsic STAT1. Our results would predict that shifts in STAT1 splicing towards a predominant expression of *Stat1*β would considerably compromise NK cell-dependent tumor surveillance and that this defect cannot be overcome by treatment with exogenous IL-15/IL-15Rα.

## Data Availability Statement

All datasets presented in this study are included in the article/Supplementary Material.

## Ethics Statement

The animal study was reviewed and approved by the Ethics and Animal Welfare Committee of the University of Veterinary Medicine Vienna and the national authority (Austrian Federal Ministry of Education, Science and Research) according to section 26ff of the Animal Experiments Act, Tierversuchsgesetz 2012 – TVG 2012 (BMWF-68.205/0218-II/3b/2012, BMWFW-68.205/0103-WF/V/3b/2015, and BMWFW-68.205/0032-WF/II/3b/2014).

## Author Contributions

KM designed and performed most of the experiments and analyzed and interpreted the data. NS, LA, AW-S, and KK performed experiments and analyzed and interpreted the data. APu helped with some experiments and analyzed the data. CL and APo provided help with *in vivo* experiments. CV helped with the statistical analysis. AD and MB provided essential materials. VS and MM were involved in the study design, provided crucial scientific input, and edited the manuscript. BS conceived the project, designed the experiments, and interpreted the data. MM, BS, and VS obtained the funding. KM and BS wrote the manuscript with input from all authors. All authors approved the manuscript.

## Conflict of Interest

The authors declare that the research was conducted in the absence of any commercial or financial relationships that could be construed as a potential conflict of interest.
