# Supplementary material for: STAT1 Isoforms Differentially Regulate NK Cell Maturation and Anti-tumor Activity
Source: Front Immunol. 2020 Sep 11;11:2189. doi: 10.3389/fimmu.2020.02189 (PMC7519029; doi:10.3389/fimmu.2020.02189)
Supplement: Supplementary file 1 [file Data_Sheet_1.pdf]

Supplementary Figure 1

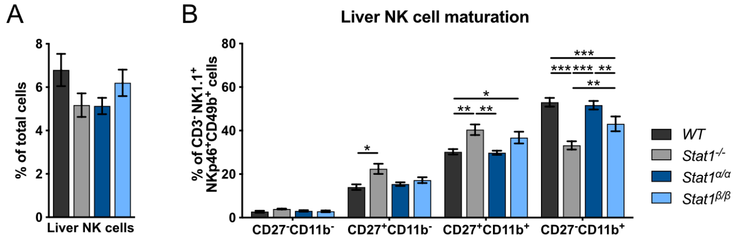

Supplementary Figure 1. *Stat1*$^{\beta/\beta}$, but not *Stat1*$^{\alpha/\alpha}$, mice show impaired liver NK cell maturation. **(A, B)** The abundance of NK cells (CD3ε$^-$NK1.1$^+$NKp46$^+$CD49b$^+$) **(A)** and NK cell maturation subsets (CD27$^-$CD11b$^-$, CD27$^+$CD11b$^-$, CD27$^+$CD11b$^+$ and CD27$^-$CD11b$^+$) **(B)** in livers from *WT*, *Stat1*$^{-/-}$, *Stat1*$^{\alpha/\alpha}$ and *Stat1*$^{\beta/\beta}$ mice were analyzed. Mean percentages ± SEM (n = 6-9) from three experiments **(A, B)** are shown. *$p < 0.05$; **$p < 0.01$; ***$p < 0.001$.

Supplementary Figure 2

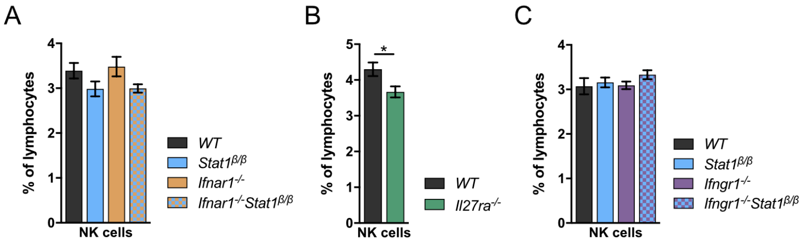

Supplementary Figure 2. The abundance of splenic NK cells is not affected by the lack of type I or type II IFN responsiveness but slightly reduced in the absence of a functional IL-27 receptor. **(A-C)** The abundance of NK cells ($CD3\varepsilon^{-}NK1.1^{+}$) in spleens from *WT*, *Stat1*$^{\beta/\beta}$ *Ifnar1*$^{-/-}$ and *Ifnar1*$^{-/-}$ *Stat1*$^{\beta/\beta}$ **(A)**, *WT* and *Il27ra*$^{-/-}$ **(B)** and *WT*, *Stat1*$^{\beta/\beta}$, *Ifngr1*$^{-/-}$ and *Ifngr1*$^{-/-}$*Stat1*$^{\beta/\beta}$ mice **(C)** was determined by flow cytometry. Mean percentages ± SEM of eight (n = 5-18) **(A)**, two (n = 12) **(B)** and six experiments (n = 10-14) **(C)** are shown. *$p < 0.05$.

Supplementary Figure 3

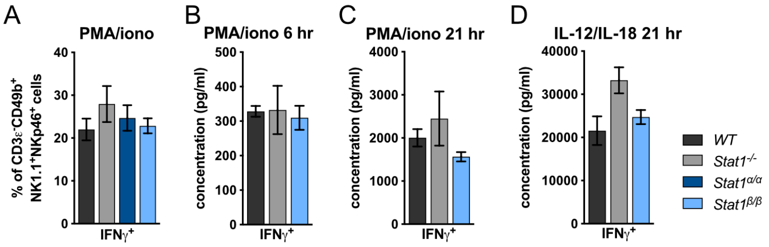

Supplementary Figure 3. *Stat1*$^{-/-}$ and *Stat1*$^{\beta/\beta}$ NK cells produce similar levels of IFNγ upon stimulation with PMA/ionomycin or IL-12/IL-18 compared to *WT* NK cells. **(A-D)** Splenocytes **(A)** and magnetic beads-purified NK cells **(B-D)** from *WT*, *Stat1*$^{-/-}$, *Stat1*$^{\alpha/\alpha}$ and *Stat1*$^{\beta/\beta}$ mice were stimulated with PMA/ionomycin **(A-C)** and IL-12 (5 ng/ml) and IL-18 (25 ng/ml) **(D)** and incubated in the presence **(A)** or absence **(B-D)** of brefeldin A for 5 hours **(A)**, 6 hours **(B)** and 21 hours **(C, D)**. IFNγ production of NK cells was analyzed by intracellular staining and flow cytometry **(A)** or in the cell culture supernatant by ELISA **(B-D)**. Mean percentages ± SEM from two experiments (n = 5-6) **(A)** and mean IFNγ concentrations ± SEM from one experiment (n = 3) **(B-D)** are depicted.

Supplementary Figure 4

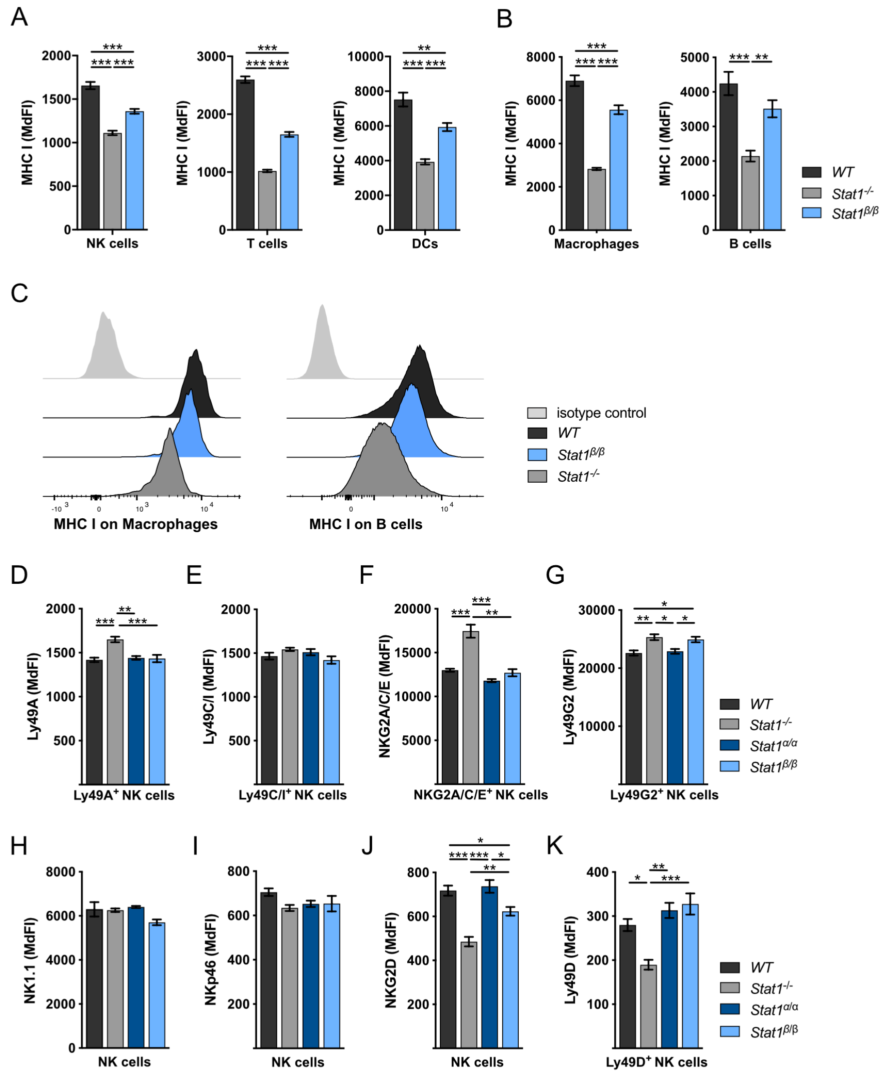

Supplementary Figure 4. In the presence of only STAT1β splenocytes have reduced MHC class I surface levels, whereas NK cells have similar inhibitory and activating receptor levels compared to *WT* cells. **(A-C)** Surface levels of MHC class I molecules on splenic NK cells, T cells, DCs, macrophages and B cells from *WT*, *Stat1*$^{-/-}$ and *Stat1*$^{\beta/\beta}$ mice were determined by flow cytometry. Quantitative analysis of surface MHC class I (MHC I) levels (MdFIs) on NK cells (left), T cells (middle) and DCs (right) **(A)**, and on macrophages (left) and B cells (right) **(B)**. Mean MdFIs ± SEM from three experiments (n = 9, **A, B** left panel and n = 5-8, **B** right panel). **(C)** Histograms show one representative sample per genotype of surface MHC class I levels on macrophages (left panel) and B cells (right panel). **(D-K)** Surface levels of the inhibitory receptors Ly49A **(D)**, Ly49C/I **(E)**, NKG2A/C/E **(F)** and Ly49G2 **(G)** and the activating receptors NK1.1 **(H)**, NKp46 **(I),** NKG2D **(J)** and Ly49D **(K)** on splenic NK cells from *WT*, *Stat1*$^{-/-}$, *Stat1*$^{\alpha/\alpha}$ and *Stat1*$^{\beta/\beta}$ mice were analysed by flow cytometry. Mean MdFIs ± SEM from two (n = 5-6) **(D-I, K)** and three (n = 8) **(J)** experiments are shown. *$p < 0.05$; **$p < 0.01$; ***$p < 0.001$.

Supplementary Figure 5

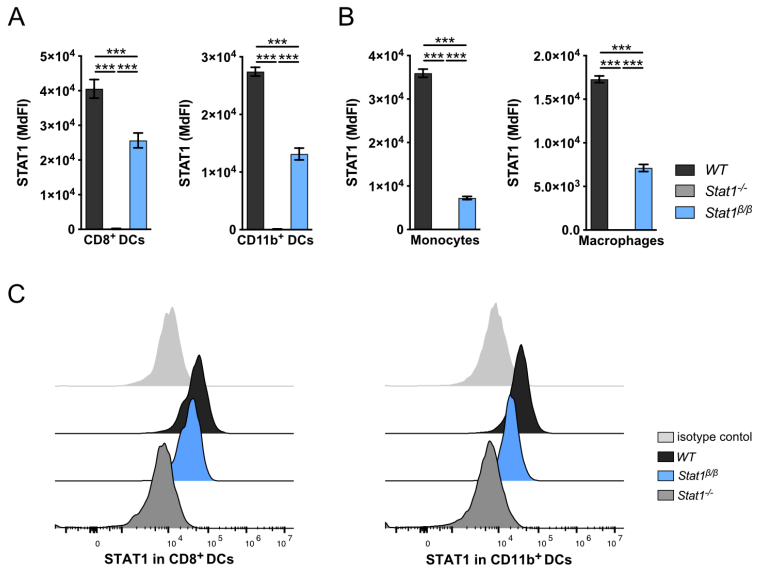

Supplementary Figure 5. *Stat1*$^{\beta/\beta}$ mice have reduced STAT1 levels in splenic DCs, monocytes and macrophages compared to *WT* cells. **(A-C)** Flow cytometry was used to determine STAT1 levels in splenic DCs subsets, monocytes and macrophages from *WT*, *Stat1*$^{-/-}$ and *Stat1*$^{\beta/\beta}$ mice. Quantitative analysis of STAT1 in CD8^{+} DCs (left) and CD11b^{+} DCs (right) **(A)** and monocytes (left) and macrophages (right) **(B)**. The average MdFI of STAT1 in *Stat1*$^{-/-}$ cells was subtracted from the MdFI of STAT1 of all samples. Mean MdFIs ± SEM (n = 6) from two experiments are shown **(A, B)**. **(C)** Histograms of one representative sample per genotype of STAT1 levels in CD8^{+} DCs (left) and CD11b^{+} DCs (right). ***$p < 0.001$.

Supplementary Figure 6

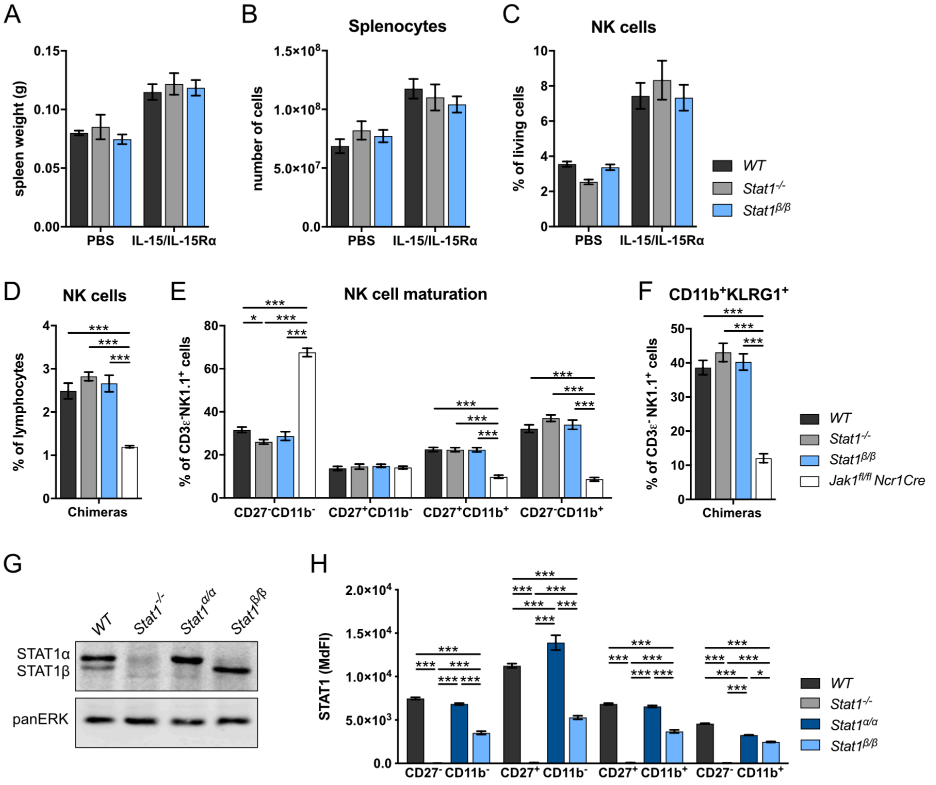

Supplementary Figure 6. IL-15 responsiveness is unaltered in NK cells from *Stat1*$^{-/-}$ and *Stat1*$^{\beta/\beta}$ and *Stat1*$^{\beta/\beta}$ NK cells exhibit normal maturation in *Jak1*$^{fl/fl}$*Ncr1Cre* bone marrow chimeras. **(A-C)** *WT*, *Stat1*$^{-/-}$ and *Stat1*$^{\beta/\beta}$ mice were treated with PBS or IL-15/IL-15Rα for one week. Spleen weight **(A)**, number of splenocytes **(B)** and percentage of NK cells **(C)** were analysed. Mean values ± SEM of three (n = 9) **(A, B)** and four experiments (n = 10-12) **(C)** are shown. **(D-F)** Splenocytes from *WT*, *Stat1*$^{-/-}$ and *Stat1*$^{\beta/\beta}$ bone marrow chimeric mice and *Jak1*$^{fl/fl}$*Ncr1Cre* controls were analyzed for the frequency of total NK cells (CD3ε^{-}NK1.1^{+}) **(D)**, the maturation subsets CD27^{-}CD11b^{-}, CD27^{+}CD11b^{-}, CD27^{+}CD11b^{+} and CD27^{-}CD11b^{+} **(E)** and CD11b^{+}KLRG1^{+} NK cells **(F)**. Mean percentages ± SEM of three experiments (n = 6-10) are shown **(D-F)**. **(G)** Splenic NK cells from *WT*, *Stat1*$^{-/-}$, *Stat1*$^{\alpha/\alpha}$ and *Stat1*$^{\beta/\beta}$ mice were FACS-sorted and STAT1 protein levels were determined by Western blot. One representative of two experiments is shown. **(H)** Total STAT1 levels were determined in NK cell maturation subsets (CD27^{-}CD11b^{-}, CD27^{+}CD11b^{-}, CD27^{+}CD11b^{+} and CD27^{-}CD11b^{+}) from *WT*, *Stat1*$^{-/-}$, *Stat1*$^{\alpha/\alpha}$ and *Stat1*$^{\beta/\beta}$ mice by flow cytometry. The average MdFI of STAT1 in *Stat1*$^{-/-}$ cells was subtracted from the MdFI of STAT1 of all samples. Mean MdFIs ± SEM (n = 6) from two experiments are depicted. **p* < 0.05; ***p* < 0.01; ****p* < 0.001.
